# Supplementary material for: Content-rich biological network constructed by mining PubMed abstracts
Source: BMC Bioinformatics. 2004 Oct 8;5:147. doi: 10.1186/1471-2105-5-147 (PMC528731; doi:10.1186/1471-2105-5-147)
Supplement: Additional File 2 — The original results of the above study (non-essential files are deleted to keep the file size under the limit set by BMC bioinformatics). [file 1471-2105-5-147-S2.bz2 › chilibotAdditionalFile2/dip10/311ID8405064E1360/html/TNFSF9_TNFRSF9.html]

 


 **TNFSF9** and **TNFRSF9** 
  
Found 93 abstracts in PubMed, retrieved 10.  
 

 What does Google say? 
 PDF only 
| .edu only 

---

**Interactive relationship** (e.g. stimulation, inhibition, etc)

**Stimulatory relationship**- 4 1BB  [ **TNFRSF9** ]  ligand  [ **TNFSF9** ]  4 1BBL  [ **TNFSF9** ] , a member of the tumor necrosis factor  [ **TNFSF9** ]  TNF superfamily, interacts with 4 1BB  [ **TNFRSF9** ]  CDw137 expressed on activated T cells and delivers a costimulatory signal for T cell activation and growth.  Ref: 12594573 Cancer Immunol Immunother, 2003
- 4 1BB  [ **TNFRSF9** ]  is a member of the tumor necrosis factor  [ **TNFSF9** ]  receptor superfamily and provides a costimulatory signal by interaction with 4 1BB  [ **TNFRSF9** ]  ligand  [ **TNFSF9** ]  expressed on antigen presenting cells.  Ref: 12706838 FEBS Lett, 2003
- An increase in the caspase activities was also observed in the HeLaTR cells transfected with 4 1BB  [ **TNFRSF9** ]  cDNA, and the 4 1BB  [ **TNFRSF9** ]  Ligand  [ **TNFSF9** ]  further increased the caspase activities of the HeLaTR cells overexpressing the 4 1BB  [ **TNFRSF9** ] .  Ref: 12846840 Eur J Biochem< MedlineTA>Eur J Biochem, 2003
**Neutral relationship**- Blocking 4 1BB  [ **TNFRSF9** ]  4 1BB  [ **TNFRSF9** ]  Ligand  [ **TNFSF9** ]  Interactions Prevents Herpetic Stromal Keratitis.  Ref: 12847221 J Immunol, 2003
- We used the murine model of HSK to demonstrate the importance of the interaction between an inducible T cell costimulatory receptor, 4 1BB  [ **TNFRSF9** ] , and its ligand, 4 1BB  [ **TNFRSF9** ]  ligand  [ **TNFSF9** ]  4 1BBL  [ **TNFSF9** ] , in the development of this disease.  Ref: 12847221 J Immunol, 2003
- In BALB c mice, HSK ordinarily induced by infection with the RE strain of herpes was prevented by blocking 4 1BB  [ **TNFRSF9** ]  4 1BBL  [ **TNFSF9** ]  interaction, either by deleting 4 1BB  [ **TNFRSF9** ]  in mutant 4 1BB  [ **TNFRSF9** ]  mice or by introducing mAbs against 4 1BBL  [ **TNFSF9** ] .  Ref: 12847221 J Immunol, 2003

**Non-interactive relationship** (e.g. studied together, co-existance, homology, etc.)

- A novel adenovirus expressing human 4 1BB  [ **TNFRSF9** ]  ligand  [ **TNFSF9** ]  enhances antitumor immunity.  Ref: 12594573 Cancer Immunol Immunother, 2003
- Provision of 4 1BB  [ **TNFRSF9** ]  ligand  [ **TNFSF9** ]  enhances effector and memory CTL responses generated by immunization with dendritic cells expressing a human tumor associated antigen.  Ref: 12626542 J Immunol, 2003
- Polyclonal CD4 T lymphocytes can be activated and expanded using anti CD3 and anti CD28 antibodies presented on magnetic beads, and CD8 T lymphocytes can be successfully expanded using a novel genetically engineered cell based technology that presents anti CD3 and anti CD28 along with the costimulatory molecule CD137  [ **TNFRSF9** ]  4 1BBL  [ **TNFSF9** ] .  Ref: 12857980 Immunol Res, 2003
- Stimulation of 4 1BBL  [ **TNFSF9** ]  on DCs with 4 1BB  [ **TNFRSF9** ]  Fc or with 4 1BB  [ **TNFRSF9** ]  transfected Jurkat cells resulted in acquisition of capacity for the immature DCs to produce interleukin 12 IL 12.  Ref: 12590704 J Hematother Stem Cell Res, 2002
- 4 1BB  [ **TNFRSF9** ]  Ligand  [ **TNFSF9** ]  Stimulation Enhances Myeloid Dendritic Cell Maturation from Human Umbilical Cord Blood CD34 Progenitor Cells.  Ref: 12590704 J Hematother Stem Cell Res, 2002
- Although treatment of the HeLaTR cells with T3 did not induce expression of NF kappaB reporter luciferase, even in the presence of the 4 1BB  [ **TNFRSF9** ]  Ligand  [ **TNFSF9** ] , it increased the caspase activities.  Ref: 12846840 Eur J Biochem< MedlineTA>Eur J Biochem, 2002
- Costimulation of human CD28 T cells by 4 1BB  [ **TNFRSF9** ]  ligand  [ **TNFSF9** ] .  Ref: 12645943 Eur J Immunol, 2003
- In this review, we will discuss the regulatory mechanisms of TNF receptor ligand family members, such as HVEM LIGHT, 4 1BB  [ **TNFRSF9** ]  4 1BBL  [ **TNFSF9** ] , and GITR GITRL that regulate T and B cell functions and participate in the process of inflammatory diseases.  Ref: 12642898 Exp Mol Med, 2003
- We investigated whether 4 1BB  [ **TNFRSF9** ]  ligand  [ **TNFSF9** ]  4 1BBL  [ **TNFSF9** ] , a member of the tumor necrosis factor  [ **TNFSF9** ]  TNF family, is involved in the maturation process to mature myeloid DCs during in vitro DC differentiation from immature DCs derived from human umbilical cord blood CB CD34 progenitor cells.  Ref: 12590704 J Hematother Stem Cell Res, 2002
